# Detecting SARS-CoV-2 From Chest X-Ray Using Artificial Intelligence

**DOI:** 10.1109/ACCESS.2021.3061621

**Published:** 2021-02-23

**Authors:** Md Manjurul Ahsan, Md Tanvir Ahad, Farzana Akter Soma, Shuva Paul, Ananna Chowdhury, Shahana Akter Luna, Munshi Md. Shafwat Yazdan, Akhlaqur Rahman, Zahed Siddique, Pedro Huebner

**Affiliations:** School of Industrial and Systems EngineeringThe University of Oklahoma 6187 Norman OK 73019 USA; School of Aerospace and Mechanical EngineeringThe University of Oklahoma 6187 Norman OK 73019 USA; Holy Family Red Crescent Medical College & Hospital Dhaka 1000 Bangladesh; School of Electrical and Computer EngineeringGeorgia Institute of Technology 1372 Atlanta GA 30332 USA; Z. H. Sikder Women’s Medical College & Hospital Dhaka 1212 Bangladesh; Dhaka Medical College & Hospital Dhaka 1000 Bangladesh; Civil and Environmental Engineering DepartmentIdaho State University 6640 Pocatello ID 83209 USA; School of Industrial Automation and Electrical EngineeringEngineering Institute of Technology 451238 Melbourne VIC 3000 Australia

**Keywords:** Artificial intelligence, COVID-19, coronavirus, SARS-CoV-2, deep learning, chest X-ray, imbalanced data, small data

## Abstract

Chest radiographs (X-rays) combined with Deep Convolutional Neural Network (CNN) methods have been demonstrated to detect and diagnose the onset of COVID-19, the disease caused by the Severe Acute Respiratory Syndrome Coronavirus 2 (SARS-CoV-2). However, questions remain regarding the accuracy of those methods as they are often challenged by limited datasets, performance legitimacy on imbalanced data, and have their results typically reported without proper confidence intervals. Considering the opportunity to address these issues, in this study, we propose and test six modified deep learning models, including VGG16, InceptionResNetV2, ResNet50, MobileNetV2, ResNet101, and VGG19 to detect SARS-CoV-2 infection from chest X-ray images. Results are evaluated in terms of accuracy, precision, recall, and f- score using a small and balanced dataset (Study One), and a larger and imbalanced dataset (Study Two). With 95% confidence interval, VGG16 and MobileNetV2 show that, on both datasets, the model could identify patients with COVID-19 symptoms with an accuracy of up to 100%. We also present a pilot test of VGG16 models on a multi-class dataset, showing promising results by achieving 91% accuracy in detecting COVID-19, normal, and Pneumonia patients. Furthermore, we demonstrated that poorly performing models in Study One (ResNet50 and ResNet101) had their accuracy rise from 70% to 93% once trained with the comparatively larger dataset of Study Two. Still, models like InceptionResNetV2 and VGG19’s demonstrated an accuracy of 97% on both datasets, which posits the effectiveness of our proposed methods, ultimately presenting a reasonable and accessible alternative to identify patients with COVID-19.

## I. Introduction

The Severe Acute Respiratory Syndrome Coronavirus 2 (SARS-CoV-2), previously known as the Novel Coronavirus, was first reported in Wuhan, China and rapidly spread around the world, pushing the World Health Organization (WHO) to declare the outbreak of the virus as a global pandemic and health emergency on March 11, 2020. According to official data, 19 million people have been infected worldwide, with the number of deaths surpassing 700, 000, and 12 million recovery cases reported by August 6, 2020 [1]. In the United States, the first case was reported on January 20, 2020, which evolved into a current number of confirmed cases, deaths, and recovered patients reaching more than 5 million, 162,000, and 2.5 million, respectively (August 6, 2020 data) [1].

COVID-19 can be transmitted in several ways. The virus can spread quickly among humans via community transmission, such as close contact between individuals, and the transfer of respiratory droplets produced via coughing, sneezing, and talking. Several symptoms have been reported so far, including fever, tiredness, and dry cough as the most common. Additionally, aches, pain, nasal congestion, runny nose, sore throat, and diarrhea have also been associated with the disease [2], [3]. Several methods can be followed to detect SARS-CoV-2 infection [4], including:

• Real-time reverse transcription polymerase chain reaction (RT-PCR)-based methods
• Isothermal nucleic acid amplification-based methods
• Microarray-based methods.

Health authorities in most countries have chosen to adopt the RT-PCR method, as it is regarded as the gold-standard in diagnosing viral and bacterial infections at the molecular level [5]. However, due to the rapidly increasing number of new cases and limited healthcare infrastructure, rapid detection or mass testing is required to lower the curve of infection. Recent studies claimed that chest Computed Tomography (CT) has the capability to detect the disease promptly. Therefore, in China, to deal with many new cases, CT scans were used for the initial screening of patients with COVID-19 symptoms [6]–[9]. Similarly, chest radiograph (X-ray) image-based diagnosis may be a more attractive and readily available method for detecting the onset of the disease due to its low cost and fast image acquisition procedure. In our study, we investigate recent literature on the topic and tackle the opportunity to present an effective deep learning-based screening method to detect patients with COVID-19 from chest X-ray images. Developing deep learning models using small image datasets often results in the incorrect identification of regions of interest in those images, an issue not often addressed in the existing literature. Therefore, in the present work, we have analyzed our models’ performance layer by layer and chose to select only the best-performing ones, based on the correct identification of the infectious regions present on the X-ray images. Also, previous works often do not demonstrate how their proposed models perform with imbalanced datasets which is often challenging. Here, we diversify the analysis and consider small, imbalanced, and large datasets while presenting a comprehensive description of our results with statistical measures, including 95% confidence intervals, $p$-values, and $t$-values. A summary of our technical contributions is presented below:

• Modification and evaluation of six different deep CNN models (VGG16, InceptionResNetV2, ResNet50, MobilenetV2, ResNet101, VGG19) for detection of COVID-19 patients using X-ray image data on both balanced and imbalanced datasets; and
• Verify the possibility to locate affected regions on chest X-rays incorporated with heatmaps, including a cross-check with a medical doctor’s opinion.

## II. Literature Review

In the recent past, the adoption of Artificial Intelligence (AI) in the field of infectious disease diagnosis has gained a notable prominence, which led to the investigation of its potential in the fight against the novel coronavirus [10]–[12]. Current AI-related research efforts on COVID-19 detection using chest CT and X-ray images are discussed below to provide a brief insight on the topic and highlight our motivations to research it further.

### A. CT Scan Based Screening

To date, several efforts in detecting COVID-19 from CT images have been reported. A recent study by Chua *et al.* (2020) suggested that the pathological pathway observed from the pneumonic injury leading to respiratory death can be detected early via chest CT, especially when the patient is scanned two or more days after the development of symptoms [13]. Related studies proposed that deep learning techniques could be beneficial for identifying COVID-19 disease from chest CT [12], [14]. For instance, Shi *et al.* (2020) introduced a machine learning-based method for the COVID-19 screening from an online COVID-19 CT dataset [15]. Similarly, Gozes *et al.* (2020) developed an automated system using artificial intelligence to monitor and detect patients from chest CT [16]. Chua *et al.* (2020) focused on the role of Chest CT in the detection and management of COVID-19 disease from a high incidence region (United Kingdom) [13]. Ai *et al.* (2020) also supported CT-based diagnosis as an efficient approach compared to RT-PCR testing for COVID-19 patients detection with a 97% sensitivity [17], [18].

Due to data scarcity, most preliminary studies considered minimal datasets [19]–[21]. For example, Chen *et al.* (2020) used a UNet++ deep learning model and identified 51 COVID-19 patients with a 98.5% accuracy [19]. However, the authors did not mention the number of healthy patients used in the study. Ardakani *et al.* (2020) used 194 CT images (108 COVID-19 and 86 other patients) and implemented ten deep learning methods to observe COVID-19 related infections and acquired 99.02% accuracy [20]. Moreover, a study conducted by Wang *et al.* (2020) considered 453 CT images of confirmed COVID-19 cases, from which 217 images were used as the training set, and obtained 73.1% accuracy, using the inception-based model. The authors, however, did not explain the model network and did not show the mark region of interest of the infections [22]. Similarly, Zheng *et al.* (2020) introduced a deep learning-based model with 90% accuracy to screen patients using 499 3D CT images [21]. Despite promising results, a very high performance on small datasets often raises questions about the model’s practical accuracy and reliability. Therefore, a better way to represent model accuracy is to present it with an associated confidence interval [23]. However, none of the work herein referenced expressed their results with confidence intervals, which should be addressed in future studies.

As larger datasets become available, deep-learning-based studies taking advantage of their potential have been proposed to detect and diagnose COVID-19. Xu *et al.* (2020) investigated a dataset of 618 medical images to detect COVID-19 patients and acquired 86.7% accuracy using ResNet23 [24]. Li *et al.* (2020) utilized an even larger dataset (a combination of 1296 COVID-19 and 3060 Non-COVID-19 patients CT images) and achieved 96% accuracy using ResNet50 [25]. With larger datasets, it is no surprise that deep learning-based models predict patients with COVID-19 symptoms with accuracies ranging from 85% to 96%. However, obtaining a chest CT scan is a notably time consuming, costly, and complex procedure. Despite allowing for comparatively better image quality, its associated challenges inspired many researchers to propose X-ray-based COVID-19 screening methods as a reliable alternative way [26], [27].

### B. Chest X-Ray Based Screening

Preliminary studies have used transfer learning techniques to evaluate COVID-19 and pneumonia cases in the early stages of the COVID-19 pandemic [28]–[31]. However, data insufficiency also hinders the ability of such proposed models to provide reliable COVID-19 screening tools based on chest X-ray [12], [32], [33]. For instance, Hemdan *et al.* (2020) proposed a CNN-based model adapted from VGG19 and achieved 90% accuracy using 50 images [32]. Ahsan *et al.* (2020) developed a COVID-19 diagnosis model using Multilayer Perceptron and Convolutional Neural Network (MLP-CNN) for mixed-data (numerical/categorical and image data). The model predicts and differentiates between 112 COVID-19 and 30 non-COVID-19 patients, with a higher accuracy of 95.4% [34]. Sethy & Behera (2020) also considered only 50 images and used ResNet50 for COVID-19 patients classification, and ultimately reached 95% accuracy [33]. Also, Narin *et al.* (2020) used 100 images and achieved 86% accuracy using InceptionResNetV 2 [12]. As noted, these studies use relatively small datasets, which does not guarantee whether their proposed models would perform equally well on larger datasets. Also, the possibility of a model overfitting is another concern for larger CNN-based networks when trained with a small datasets.

In view of these issues, recent studies proposed model training with larger datasets and reported a better performance compared to smaller ones [35]–[38]. Chandra *et al.* (2020) developed an automatic COVID screening system to detect infected patients using 2088 (696 normal, 696 pneumonia, and 696 COVID-19) and 258 (86 images of each category) chest X-ray images, and achieved 98% accuracy [39]. Sekeroglu *et al.* (2020) developed a deep learning-based method to detect COVID-19 using publicly available X-ray images (1583 healthy, 4292 pneumonia, and 225 confirmed COVID-19), which involved the training of deep learning and machine learning classifiers [40]. Pandit *et al.* (2020) explored pre-trained VGG-16 using 1428 chest X-rays with a mix of confirmed COVID-19, common bacterial pneumonia, and healthy cases (no infection). Their results showed an accuracy of 96% and 92.5% in two and three output class cases [41]. Ghosal & Tucker (2020) used 5941 chest X-ray images and obtained 92.9% accuracy [11]. Brunese *et al.* (2020) proposed a modified VGG16 model and achieved 99% accuracy with a dataset of 6505 images. However, they have used fairly balanced data with a 1:1.17 ratio; 3003 COVID-19 and 3520 other patients. It is not immediately clear how their model would perform on an imbalanced dataset [42]. On the other hand, Khan *et al.* (2020) developed a model based on Xception CNN techniques considering 284 COVID-19 patients and 967 other patients (data ratio 1:3.4). Partially as an effect of a more imbalanced dataset, their reported accuracy was comparatively low, reaching 89.6% [38]. On imbalanced datasets, there is a higher chance that the model may be biased on significant classes and might affect the overall performance of the model.

## III. Research Methodology

We propose three separate studies, wherein three distinct datasets were used, as detailed below:

1) Study One – smaller, balanced dataset: chest X-ray images of 25 patients with COVID-19 symptoms, and 25 images of patients with diagnosed pneumonia, obtained from the open-source repository shared by Dr. Joseph Cohen [43].
2) Study Two – larger, imbalanced dataset: chest X-ray images of 262 patients with COVID-19 symptoms, and 1583 images of patients with diagnosed pneumonia, obtained from the Kaggle COVID-19 chest X-ray dataset [44].
3) Study Three – multiclass dataset: chest X-ray images of 219 patients with COVID-19 symptoms, 1345 images of patients with diagnosed pneumonia and 1073 images of normal patients, also obtained from the Kaggle COVID-19 chest X-ray dataset [45].

**FIGURE 1.**
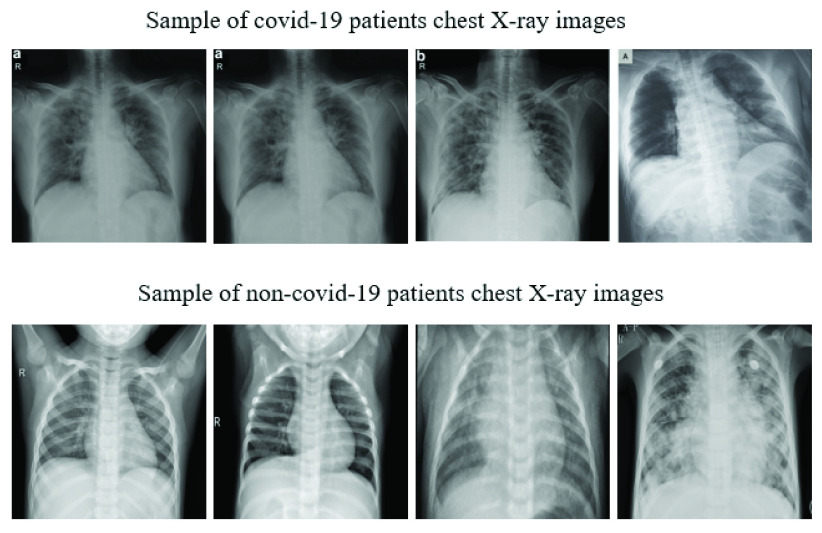
Representative samples of chest X-ray images from the open source data repositories [43] used in our proposed studies.

Figure 1 presents a set of representative chest X-ray images of both COVID-19 and pneumonia patients from the aforementioned datasets. Table 1 details the overall assignment of data for training and testing of each investigated CNN model. In both studies, six different deep learning approaches were investigated: VGG16 [46], InceptionResNetV2 [47], ResNet50 [48], MobileNetV2 [49], ResNet101 [50] and VGG19 [46].

**TABLE 1:** Assignment of Data Used for Training and Testing of Deep Learning Models

Study	Label	Train	Test
One	COVID-19	20	5
	Normal	20	5
Two	COVID-19	210	52
	Normal	1266	317
Three	COVID-19	176	43
	Normal	1073	268
	Pneumonia	1076	269

### A. Using Pre-Trained Convet

**FIGURE 2.**
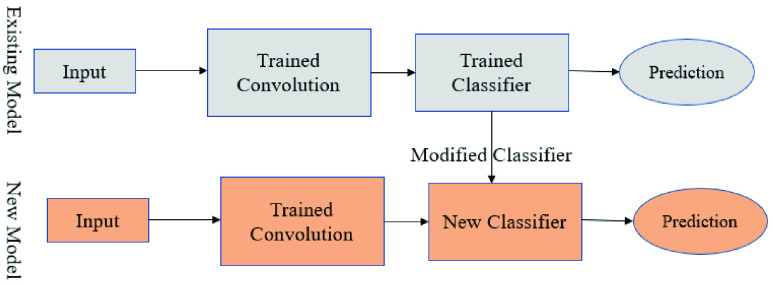
Modified architecture with new classifier [51].

A pre-trained network is a network that was previously trained on a larger dataset which, in most cases, is enough to learn a unique hierarchy to extract features from. It works more effectively on small datasets. A prime example is the VGG16 architecture, developed by Simoyan and Zisserman (2014) [51]. Figure 2 shows a sample architecture of the pre-trained model procedure. All models implemented in this study are available as a pre-package within Keras [51].

**FIGURE 3.**
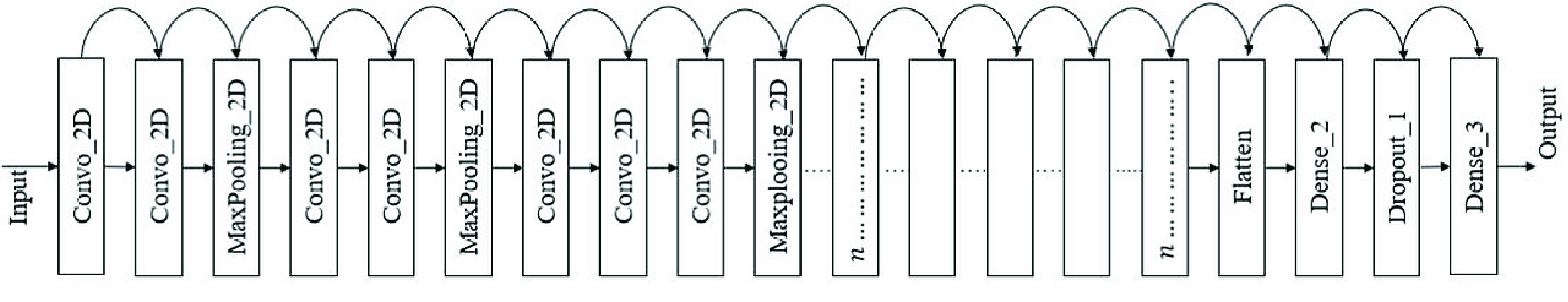
VGG16 architecture used during this experiment.

Figure 3 demonstrates a fine-tuning sequence on the VGG16 network. The modified architecture follows the steps below:

1) Firstly, the models were initiated with a pre-trained network without a fully connected (FC) layer.
2) Then, an entirely new connected layer added a pooling layer and “softmax” as an activation function, appended it on top of the VGG16 model.
3) Finally, the convolution weight was frozen during the training phase so that only the FC layer should train during the experiment.

The same procedure was followed for all other deep learning techniques. In this experiment, the additional modification of the model for all CNN architectures was constructed as follows: $AveragePooling2D(Pool size=(4,4))\rightarrow Flatten\rightarrow Dense \rightarrow Dropout (0.5) \rightarrow Dense (Activation=``softmax'')$. As it is known, most pre-trained models contain multiple layers which are associated with different parameters (i.e., number of filters, kernel size, number of hidden layers, number of neurons) [52]. However, manually tuning those parameters is considerably time consuming [53], [54]. With that in mind, in our models, we have optimized three parameters: batch size,^1^ epochs,^2^ and learning rate ^3^ (inspired by [57], [58]). We used the grid search method [59], which is commonly used for parameter tuning. Initially, we randomly selected the following:

\begin{align*} \text {Batch}\, \text {size}=&[{4,5,8,10}]\\ \text {Number}\, \text {of}\, \text {epochs}=&[{10,20,30,40}]\\ \text {Learning}\, \text {rate}=&[.001,.01, 0.1]\end{align*}

^1^Batch size characterizes the number of samples to work through before updating the internal model parameters [55]^2^It defines how many times the learning algorithm will work through the entire dataset [55]^3^It is a hyper-parameter that controls the amount to change the model in order to calculate the error each time the model weights are updated [56]

For Study One, using the grid search method, we achieved better results with the following:

\begin{align*} \text {Batch}\, \text {size}=&8\\ \text {Number}\, \text {of}\, \text {epochs}=&30\\ \text {Learning}\, \text {rate}=&.001\\{}\end{align*}

Similarly, for Study Two, the best results were achieved with:

\begin{align*} \text {Batch}\, \text {size}=&50\\ \text {Number}\, \text {of}\, \text {epochs}=&50\\ \text {Learning}\, \text {rate}=&.001\\{}\end{align*}

Finally, during Study Three, best performance was achieved with:

\begin{align*} \text {Batch}\, \text {size}=&50\\ \text {Number}\, \text {of}\, \text {epochs}=&100\\ \text {Learning}\, \text {rate}=&.001\\{}\end{align*}

We used the adaptive learning rate optimization algorithm (Adam) as an optimization algorithm for all models due to its robust performance on binary image classification [60], [61]. As commonly adopted in data mining techniques, this study used 80% data for training, whereas the remaining 20% was used for testing [62]–[64]. Each study was conducted twice, and the final result was represented as the average of those two experiment outcomes, as suggested by Zhang *et al.* (2020) [65]. Performance results were presented as model accuracy, precision, recall, and f-score [66].

\begin{align*} \textrm {Accuracy}=&\frac {t_{p} + t_{n}}{t_{p} + t_{n} + f_{p} + f_{n}} \tag{1}\\ \textrm {Precision}=&\frac {t_{p}}{t_{p}+f_{p}} \tag{2}\\ \textrm {Recall}=&\frac {t_{p}}{t_{n}+f_{p}} \tag{3}\\ \textrm {F-score}=&2\times \frac {\textrm {Precision}\times \textrm {Recall}}{\textrm {Precision+Recall}}\tag{4}\end{align*}

where,

• True Positive ( $t_{p}$) = COVID-19 patient classified as patient
• False Positive ( $f_{p}$) = Healthy people classified as patient
• True Negative ( $t_{n}$) = Healthy people classified as healthy
• False Negative ( $f_{n}$) = COVID-19 patient classified as healthy.

## IV. Results

### A. Study One

The overall model performance for all CNN approaches was measured both on the training (40 images) and test (10 images) sets using equation 1, 2, 3, and 4. Table 2 presents the results of the training set. In this case, VGG16 and MobileNetV2 outperformed all other models in terms of accuracy, precision, recall, and f score. In contrast, the ResNet50 model showed the worst performance across all measures.

**TABLE 2:** Study One Model Performance on Train Set

Model	Accuracy	Precision	Recall	F-score
VGG16	100%	100%	100%	100%
InceptionResNetV2	97%	98%	97%	97%
ResNet50	70%	81%	70%	67%
MobileNetV2	100%	100%	100%	100%
ResNet101	80%	83%	80%	80%
VGG19	93%	93%	93%	92%

Table 3 presents the performance results for all models on the test set. Models VGG16 and MobileNetV2 showed 100% performance across all measures. On the other hand, ResNet50, ResNet101, and VGG19 demonstrated significantly worse results.

**TABLE 3:** Study One Model Performance on Test Set

Model	Accuracy	Precision	Recall	F-score
VGG16	100%	100%	100%	100%
InceptionResNetV2	97%	98%	97%	97%
ResNet50	70%	81%	70%	67%
MobileNetV2	100%	100%	100%	100%
ResNet101	70%	81%	70%	67%
VGG19	70%	71%	70%	70%

#### 1) Confusion Matrix

**FIGURE 4.**
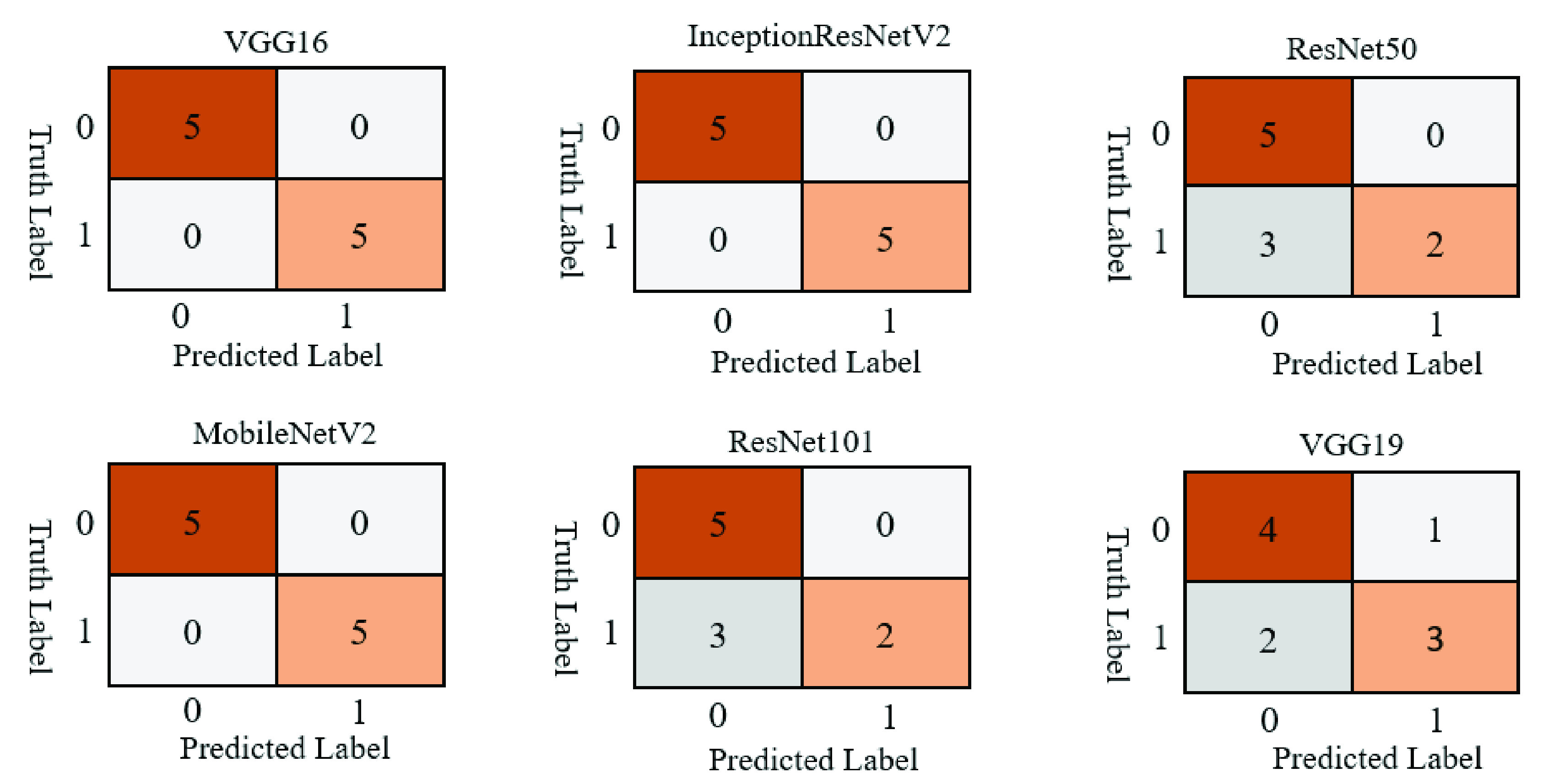
Study one confusion matrices for six different deep learning models applied on the test set.

Confusion matrices were used to better visualize the overall performance of prediction. The test set contains 10 samples (5 COVID-19 and 5 other patients). In accordance with the performance results previously presented, Figure 4 shows that the VGG16, InceptionResNetV2, and MobileNetV2 models correctly classified all patients. In contrast, models ResNet50, and ResNet101 incorrectly classified 3 non-COVID-19 patients as COVID-19 patients, and models VGG19 classified 2 non-COVID patients as COVID-19 patients while also classifying 1 COVID-19 patient as non-COVID-19.

#### 2) Model Accuracy

**FIGURE 5.**
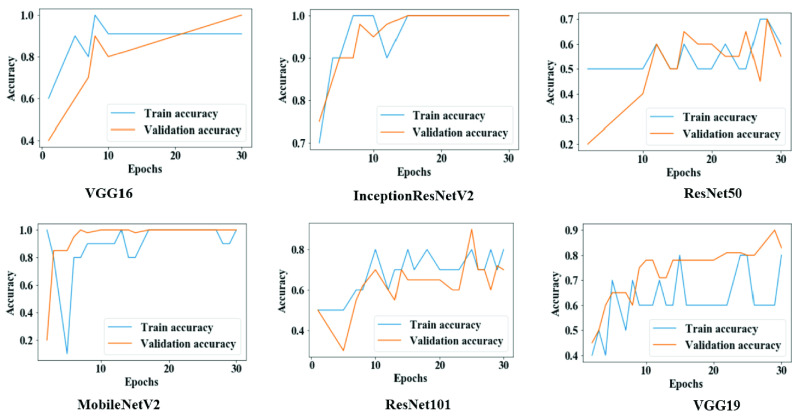
Training and validation accuracy throughout the execution of each model in study one.

Figure 5 shows the overall training and validation accuracy during each epoch for all models. Models VGG16 and MobileNetV2 demonstrated higher accuracy at epochs 25 to 30, while VGG19, ResNet50, and ResNet101 displayed lower accuracy which sporadically fluctuated between epochs 10.

#### 3) Model Loss

**FIGURE 6.**
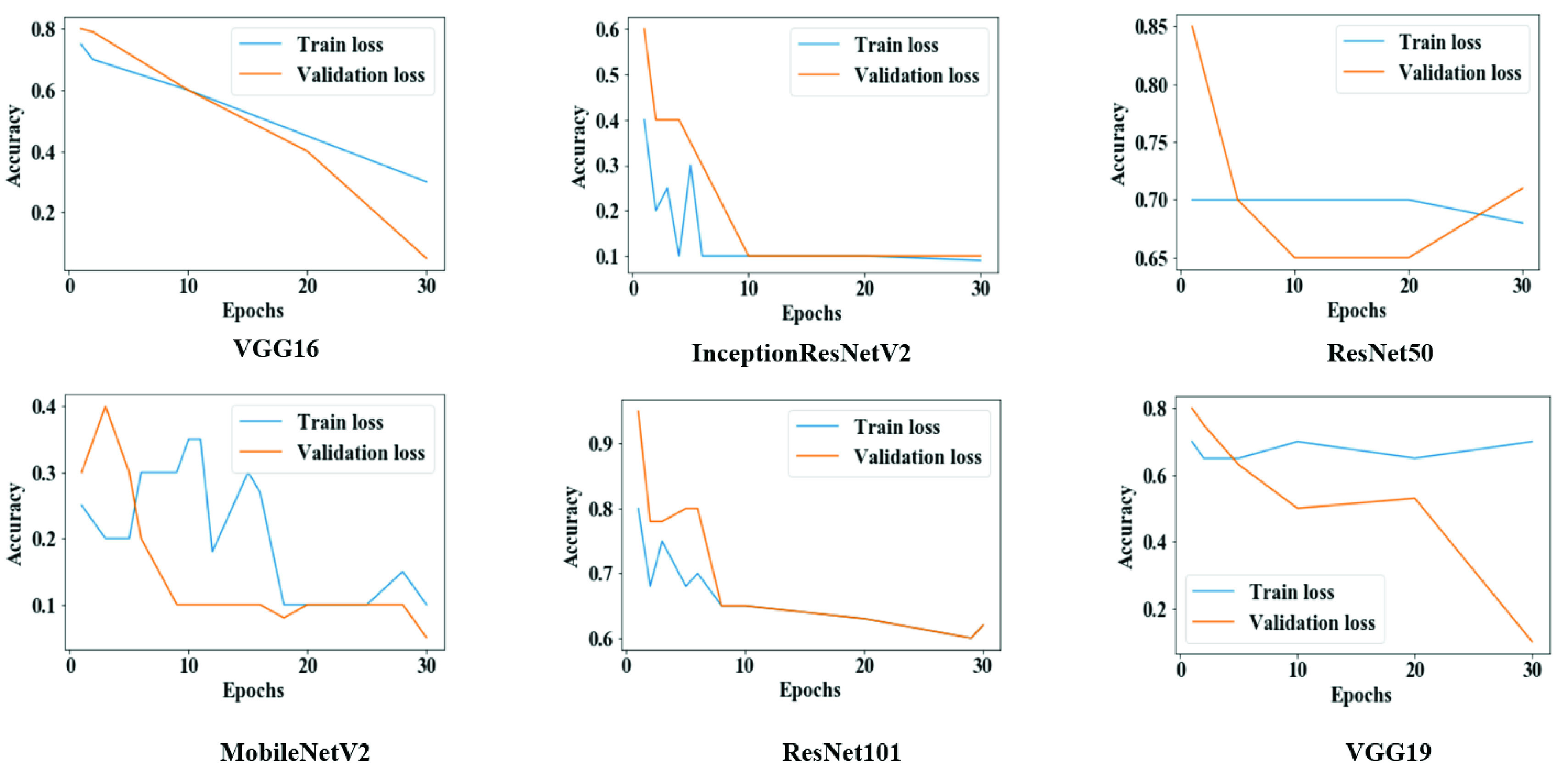
Training and validation loss throughout the execution of each model in study one.

Figure 6 shows that both training loss and validation loss were reduced following each epoch for VGG16, InceptionResNetV2, and MobileNetV2. In contrast, for VGG19, both measures are scattered over time, which is an indicative of poor performance.

### B. Study Two

For Study Two, on the training set, most model accuracies were measured above 90%. Table 4 shows that 100% accuracy, precision, recall, and f score were achieved using MobileNetV2. Among all other models, ResNet50 showed the worst performance across all measures.

**TABLE 4:** Study Two Model Performance on Train Set

Model	Accuracy	Precision	Recall	F-score
VGG16	99%	99%	99%	99%
InceptionResNetV2	99%	100%	99%	99%
ResNet50	93%	96%	93%	93%
MobileNetV2	100%	100%	100%	100%
ResNet101	96%	97%	88%	92%
VGG19	99%	98%	96%	97%

Table 5 presents the performance results for all models on the test set. Models VGG16, InceptionResNetV2, and MobileNetV2 showed 99% accuracy; however, the precision, recall, and f score were distinct for each model, yet all above 97%. On the lower end, ResNet50 demonstrated relatively lower performance across all measures.

**TABLE 5:** Study Two Model Performance on Test Set.

Model	Accuracy	Precision	Recall	F-score
VGG16	99%	100%	97%	98%
InceptionResNetV2	99%	99%	98%	98%
ResNet50	93%	94%	93%	92%
MobileNetV2	99%	99%	99%	99%
ResNet101	96%	97%	88%	92%
VGG19	97%	97%	91%	94%

#### 1) Confusion Matrix

**FIGURE 7.**
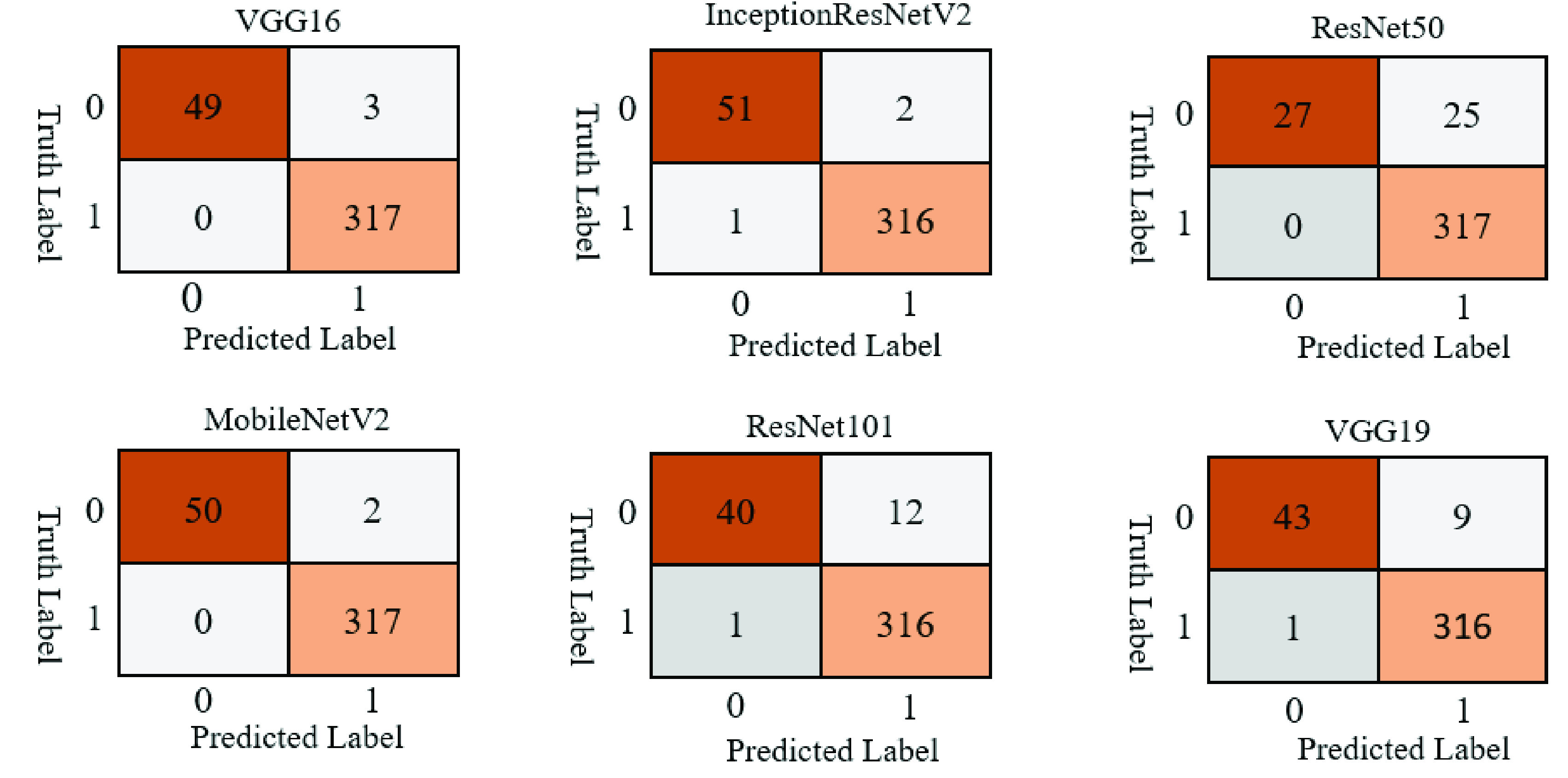
Study two confusion matrices for six different deep learning models applied on the test set.

Figure 7 shows that most of the models performance is satisfactory on the test set. In Study Two, classification accuracy for ResNet50 and ResNet101 is significantly better compared to Study One, possibly as an effect of the models being trained with more data and more epochs. In general, MobileNetV 2 performed better among all the models and misclassified only 2 images out of 369 images, while ResNet50 showed lower performance and misinterpreted 25 images out of 369 images.

#### 2) Model Accuracy

**FIGURE 8.**
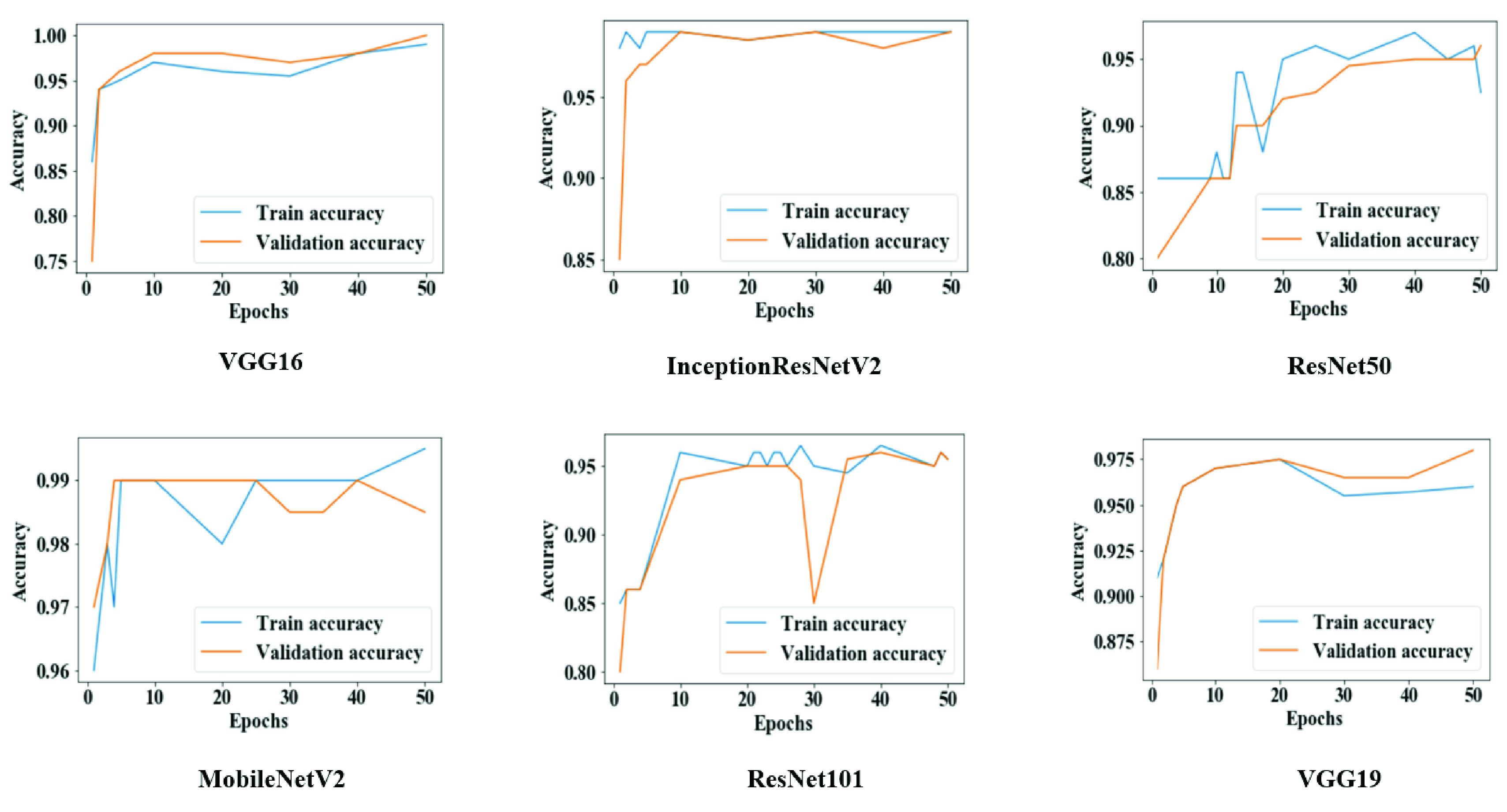
Training and validation accuracy throughout the execution of each model in study two.

Figure 8 suggests that the overall training and validation accuracy were more steady during Study Two than Study One. The performance of ResNet50 and ResNet101 significantly improved once trained with more data (1845 images) and more epochs (50 epochs).

#### 3) Model Loss

**FIGURE 9.**
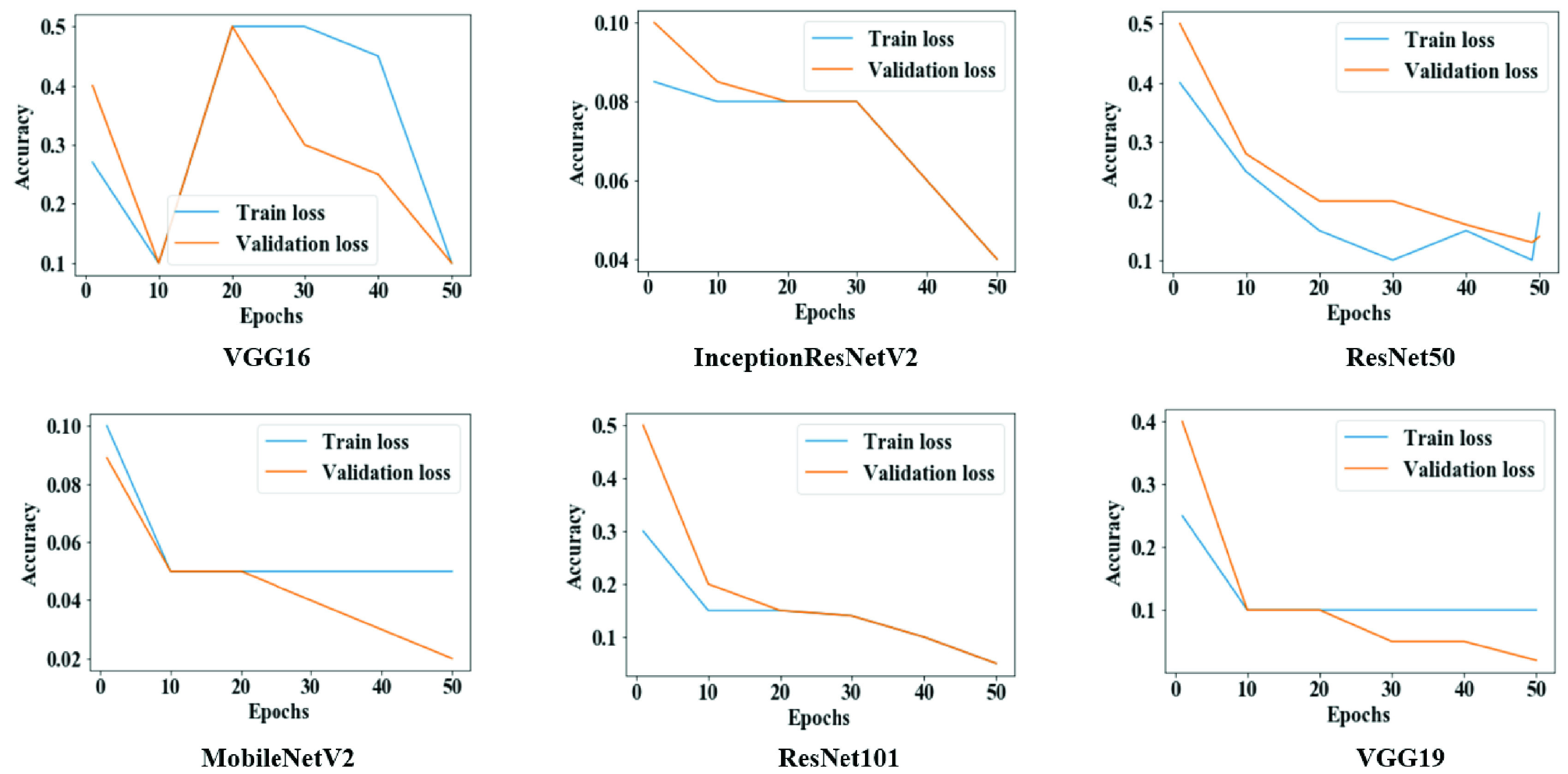
Training and validation loss throughout the execution of each model in study two.

Figure 9 provides evidence that both training and validation losses were minimized following each epoch for all models, potentially as an effect of the increased batch size, number of epochs, and data amount.

### C. Study Three

As means of highlighting the potential of our proposed models with more complex classifications, we executed a small-scale pilot study to assess the performance of the VGG16 model on a multi-class dataset. The performance outcomes for the train and test runs are presented in Table 6. The accuracy remained above 90% on both runs, which suggests a notably high performance of our model with either binary or multi-class datasets.

**TABLE 6:** VGG16 Model Performance on Train and Test Datasets of Study Three

VGG16	Accuracy	Precision	Recall	F-score
Train	93%	93%	88%	91%
Test	91%	90%	91%	86%

### D. Test Results With Confidence Intervals

Table 7 presents 95% confidence intervals for model accuracy on the test sets for Studies One and Two. For instance, in Study One, the average accuracies for VGG16 and MobileNetV2 were found to be 100%; however, the Wilson score and Bayesian interval show that the estimated accuracies lie between 72.2% to 100% and 78.3% to 100%, respectively. On the other hand, Study Two reported relatively narrower interval ranges.

**TABLE 7:** Confidence Interval ( $\alpha= 0.05$) for Studies One and Two on Test Accuracy

Study	Model	Actual accuracy	Methods	
			Wilson Score	Bayesian Interval
One	VGG16	100%	72.2% – 100%	78.3% – 100%
	InceptionResNetV2	97%	68.1% – 99.8%	72.5% – 99.9%
	ResNet50	70%	39.7% – 89.2%	39.4% – 90.7%
	MobileNetV2	100%	72.2% – 100%	78.3% – 100%
	ResNet101	70%	39.7% – 89.2%	39.4% – 90.7%
	VGG19	70%	39.7% – 89.2%	39.4% – 90.7%
Two	VGG16	99%	97.6% – 99.7%	97.8% – 99.8%
	InceptionResNetV2	99%	98.0% – 99.9%	98.3% – 99.9%
	ResNet50	93%	90% – 95.4%	90.3% – 95.5%
	MobileNetV2	99%	97.6% – 99.7%	97.8% – 99.8%
	ResNet101	96%	94.1% – 97.9%	94.2% – 98.0%
	VGG19	97%	95.1% – 98.5%	95.2% – 98.6%

A paired t-test was conducted to compare model accuracies on both studies as shown in Table 8. There was no significant difference identified within the scores for Study One ( $M = 84.50$, SD = 15.922) and Study Two ( $M = 97.39$, SD = 2.38); $t(5) = -2.251$, $p =.074$. These results suggest that model accuracy is competent on both datasets and makes no statistically significant differences ( $p>0.05$).

**TABLE 8:** Descriptive Statistics of Paired t-Test for Study One and Study Two. $M$ – Mean; SD – Standard Deviation; SEM – Standard Error Mean; DF – Degree of Freedom

Study	M	SD	SEM
One	84.50	15.922	6.50
Two	97.3967	2.38362	.9731
Paired Difference	−12.89	14.03	5.72
Results			
t	−2.251		
DF	5		
$\alpha =.05$	.074		

## V. Discussion

As a means of comparing our results with those available in the literature, Table 9 contrasts the accuracies of our three best performing CNN models on small datasets as part of Study One. It is relevant to emphasize that none of the referenced studies presents their results as confidence intervals, which hinders a direct comparison, but still allows for a higher-level assessment of the reported performance measures.

**TABLE 9:** Different Deep CNN Models Performance on Small Chest X-Ray Image Dataset

Reference	Model	Datasize	Accuracy
Hemdan et al. [32]	VGG19	50	90%
Sethy & Behera [33]	ResNet50+SVM	50	95%
Narin et al. [12]	InceptionResNetV2	100	86%
Best model from Study One with 95% CI	VGG16 InceptionResNetV2 MobileNetV2	50	72.2% – 100%
			68.1% – 99.8%
			72.2% – 100%

Using 50 chest X-ray images, we have achieved accuracy ranges from 68.1% to 99.8% using InceptionResNetV2, while Narin *et al.* (2020) used 100 images and obtained 86% accuracy [12]. Hemdan *et al.* (2020) and Sethy & Behera (2020) used small datasets of 50 images and acquired 90% and 9% accuracy using VGG19 and ResNet50+ SVM, respectively [32], [33].

Additionally, In Study Two, some of our models—VGG16, InceptionResNetV2, MobileNetV2,VGG19— demonstrated almost similar accuracy while considering a highly imbalanced dataset than referenced literature [37], [38] that also used imbalanced datasets (Table 10). For the imbalanced dataset, we used 262 COVID-19 and 1583 non-COVID-19 patients’ (1:6.04) chest X-ray images. Apostolopoulos and Mpesiana (2020) used 1428 chest X-ray images where the data ratio was 1:5.4 (224 COVID-19: 1208 others) and achieved 98% accuracy [36]. Similarly, Khan *et al.* (2020) used 1251 chest X-ray images, data proportion 1:3.4 (284 COVID-19:967 others), and acquired 89.6% accuracy [38]. In Study Two, some of the best models we acquired were VGG16, VGG19, InceptionResNetV2, and MobileNetV2 and accuracy lies between 97% to around 100%.

**TABLE 10:** Comparison of Models Performance on Imbalanced Datasets

Reference	Model	Datasize	Data ratio	Accuracy
Brunese et al. [42]	VGG16	6505	3003: 3520 ≈ 1: 1.17	99%
Apostolopoulos & Mpesiana [36]	CNN	1428	224: 1208 ≈ 1: 5.4	98%
Ozturk et al. [37]	DarkNet	1750	250: 1500=1: 6	98%
Khan et al. [38]	Xception	1251	284: 967 ≈ 1: 3.4	89.6%
Lee et al. [67]	VGG-16	1821	607: 1214=1: 2	95.9%
Shelke et al. [68]	DenseNet-VGG16	2271	500: 1771 ≈ 1: 3.54	95%
Best model from Study Two with 95% CI	VGG 16 InceptionResNetV2 MobileNetV2 VGG 19	1845	262: 1583≈1:6.04	97.6%–99.7%
				97.6%–99.7%
				97.6%–99.7%
				95.2%–98.6%

### A. Feature Selection

**FIGURE 10.**
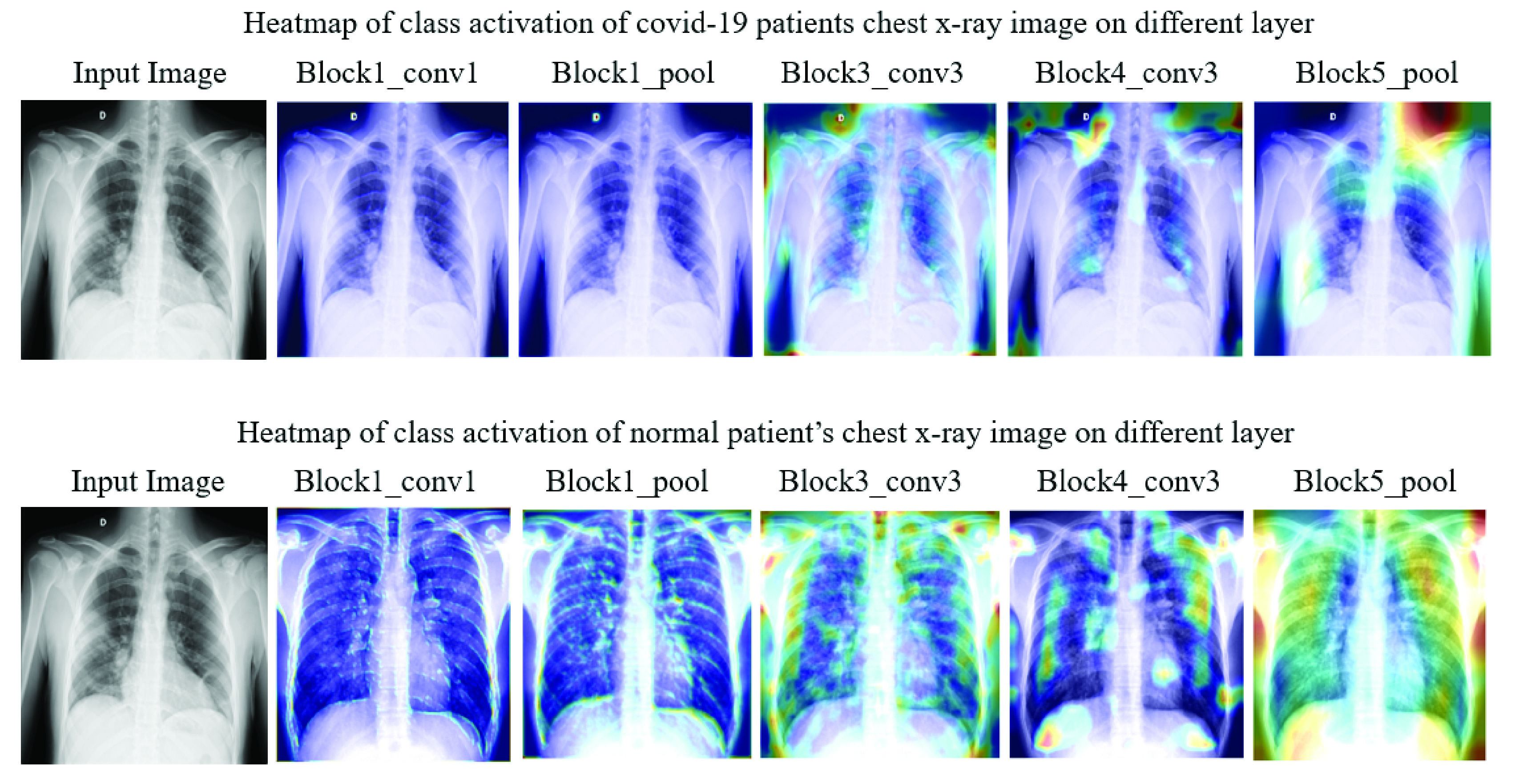
Heatmap of class activation on different layers.

**FIGURE 11.**
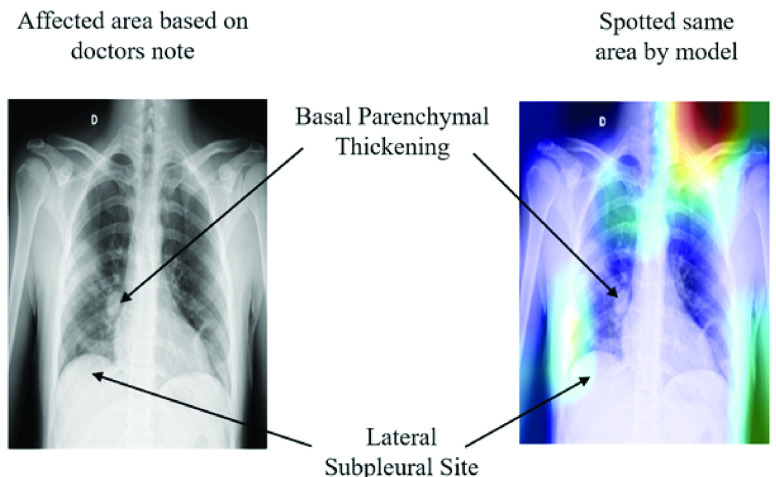
Model’s ability to identify important features on chest X-ray using VGG16.

Figure 10 highlights extracted features as an effect of different CNN layers of VGG16 models applied on chest X-ray images from Study One. For instance, in block1_conv1 and block1_pool1, the extracted features were slightly fuzzy, while in block4_conv3 and block5_pool, those features become more visible/prominent. The heatmap also demonstrates a considerable difference in both COVID-19 and other patient images corresponding to each layer. For instance, as shown in Figure 11 (left), two specific regions were highlighted by heatmap for the COVID-19 patient’s X-ray image, whereas for other patients’ images, the areas were found to be haphazard and small.

During the experiment, each layer plays a significant role in identifying essential features from the images in the training phase. As a result, it is also deemed possible to see which features are learned and play a crucial role in differentiating between the two classes. In Figure 11, the left frame represents a chest X-ray image of a COVID-19 patient, and the right one highlights infectious regions of that same image, as spotted by the VGG16 model during Study One. The highlighted region on the upper right shoulder, which resulted from the individual layer of the VGG16 model (Study One), can be considered an irrelevant and therefore unnecessary feature identified by the network. The following topics extend the discussion on this issue:

1) The models attained unnecessary details from the images since the dataset is small compared to the model architecture (contains multiple CNN layers).
2) The models extracted features beyond the center of the images, which might not be essential to differentiate the COVID-19 patients from the non-COVID-19 patients.
3) The average age of COVID-19 patients in the first case study is 55.76 years. Therefore it is possible that individual patients might have age-related illnesses (i.e. weak/damaged lungs, shoulder disorder), apart from complications related to COVID-19, which are not necessarily considered by the doctor’s notes.

**FIGURE 12.**
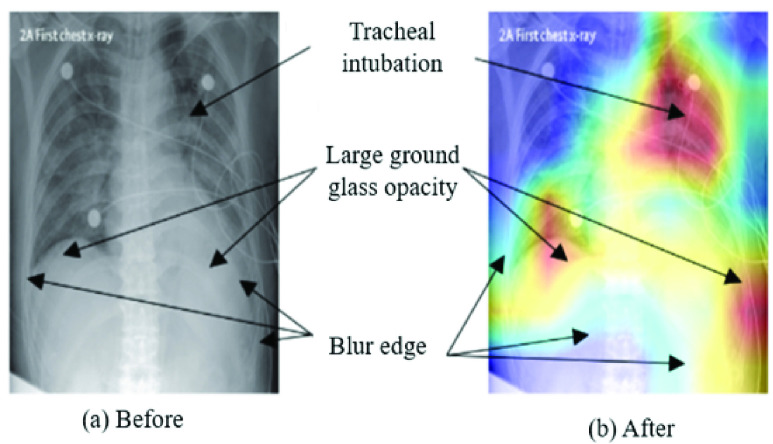
Model’s competency to identify essential features on chest X-ray using MobileNetV2.

Interestingly, the these irrelevant regions spotted by our models decreased significantly when trained with larger datasets (1845 images) and increased epochs (50 epochs). For instance, Figure 12, presents the heatmap of the Conv-1 layer of MobileNetV2, acquired during the Study Two. The heatmap verifies that the spotted regions are very similar and match closely with the doctor’s findings.

## VI. Limitations of the Study

We present the following items as limitations of our study, which shall be addressed in future works that consider our choice of tools and methods:

• At the time of writing, the limited availability of data represented a challenge to confidently assess the performance of our models. Open databases of COVID-19 patient records, especially those containing chest X-ray images, are rapidly expanding and should be considered in ongoing and future studies.
• We did not consider categorical patient data such as age, gender, body temperature, and other associated health conditions that are often available in medical datasets. More robust classification models that use those variables as inputs should be investigated as a means of achieving higher performance levels.
• We were limited to assessing the classification performance of our models against the gold standards of COVID-19 testing. However, those gold standards themselves are imperfect and often present false positives/negatives. It is imperative to ensure that the training sets of AI models like those herein presented are classified to the highest standards.
• Lastly, our study did not explore the compatibility of our proposed models with existing computer-aided diagnosis (CAD) systems. From a translational perspective, future works should explore the opportunity to bridge that gap with higher priority.

## VII. Conclusion and Future Works

Our study proposed and assessed the performance of six different deep learning approaches (VGG16, InceptionResNetV2, ResNet50, MobileNetV2, ResNet101, and VGG19) to detect SARS-CoV-2 infection from chest X-ray images. Our findings suggest that modified VGG16 and MobileNetV2 models can distinguish patients with COVID-19 symptoms on both balanced and imbalanced dataset with an accuracy of nearly 99%. Our model outputs were crosschecked by healthcare professionals to ensure that the results could be validated. We hope to highlight the potential of artificial-intelligence-based approaches in the fight against the current pandemic using diagnosis methods that work reliably with data that can be easily obtained, such as chest radiographs. Some of the limitations associated with our work can be addressed by conducting experiments with extensively imbalanced big data, comparing the performance of our methods with those using CT scan data and/or other deep learning approaches, and developing models with explainable artificial intelligence on a mixed dataset.
